# The effective treatment of dye-containing simulated wastewater by using the cement kiln dust as an industrial waste adsorbent

**DOI:** 10.1038/s41598-024-64191-5

**Published:** 2024-06-25

**Authors:** Eslam Syala, Wagih A. Sadik, Abdel-Ghaffar M. El-Demerdash, Waffa Mekhamer, M. Essam El-Rafey

**Affiliations:** Department of Materials Science, Institute of Graduate Studies and Researches (IGSR), Alexandria University, 163 Horreya Avenue, Shatby, 21526 Alexandria Egypt

**Keywords:** Wastewater, Methylene blue, Congo red, Dyes, Cement kiln dust, Environmental impact, Pollution remediation

## Abstract

This study compares the adsorption behavior of both Methylene Blue (MB) and Congo Red (CR) dyes on the surfaces of cement kiln dust (CKD) powder from the experimentally simulated wastewater solution. The cement kiln dust powder was characterized using X-ray Fluorescence (XRF), X-ray diffraction (XRD), N_2_ adsorption–desorption Brunauer–Emmett–Teller (BET), Fourier Transform Infrared Spectroscopy (FTIR), and Scanning Electron Microscopy (SEM) tests. The adsorption for such dyes was studied under varying mixing contact times, temperatures, and pH as well as various initial concentrations of both dyes and adsorbent using the batch mode experiments. Pseudo-first-order, pseudo-second-order, and intraparticle diffusion models were applied, and the results revealed that the pseudo-second-order fitted well to the kinetic data. Thermodynamic parameters stated that the adsorption process was endothermic. Studying Linear and nonlinear forms of Langmuir and Freundlich's adsorption isotherms revealed that the adsorption process was followed by both homogeneous mono-layer and heterogeneous multilayer coverage on the active sites of cement kiln dust particles. The data showed that the adsorption capacities of the methylene blue and Congo red dyes were 58.43 and 123.42 mg/g, respectively and cement kiln dust is an adsorbent with little cost for the treatment of wastewater.

## Introduction

Dyes (either natural or synthetic) are one of the most consumable coloring agents that are used in various industries like paint, clothes, cosmetics, plastics, leather, rubber, and food. The use of natural dyes in the coloring process is known to reduce the environmental and health impacts rather than synthetic dyes^1^. Although efforts have been made to produce natural dyes to be used in various industries, synthetic dyes are still manufactured and used with more than 10,000 tons globally^2^. The textile industry is one of the industries that consumes large amounts of water and produces large amounts of dyes, approximately 200,000 tons yearly, which are considered pollutants. These amounts of dye molecules are finally carried out on water, making the dyes one of the sources of water pollution^3^. This wastewater is discharged into the surrounding environment causing serious soil and water pollution. These pollutants carrying wastewater can be taken up by human beings, or animals out of the food chain causing fatal diseases such as cancer, skin irritation, mutagenic changes, delayed nervous responses, neurological disorders, etc.^4^. Therefore, these large amounts of wastewater should be treated to remove the largest proportion of pollutants that can be removed and meet both national and international standards. There are numerous methods, namely chemical, physical, and biological methods, that can be used to treat wastewater. Although conventional biological methods are eco-friendly, cost-effective, and broadly applied in the past, they are ineffective in removing the color of resistant dyes^5^. Both physical and chemical methods are more suitable and efficient for this purpose. These methods include ion exchange, precipitation, electrocoagulation, adsorption, oxidation, evaporation, reverse osmosis, electrochemistry, membrane filtration, Fenton oxidation, bio-degradation, ozonation and phytoremediation^6,7^. Among these methods, adsorption is at the top of these techniques because it is found to be a cheap, eco-friendly, and active technique for the elimination of dyes from industrial wastewater^8^. It is a surface concept that concerns the charges/surface forces among the dye, the medium (water), and the adsorbent (solid material that absorbs the dye). The adsorbents can generally be driven from industrial wastes either from natural or agricultural sources. On the other hand, dyes are classified into cationic, anionic, and nonionic dyes^9^. Methylene blue (MB) dye was found to be the most commonly used dye in the textile industry. It is carcinogenic, toxic, and non-biodegradable that can create a stable solution with water at ambient temperature^10^. Besides its use in the textile industry, MB is also used in medical applications such as a staining agent and the treatment of methemoglobinemia and cyanide poisoning^11^. Long exposure to it can cause serious health problems such as Jaundice, abdominal disorders, tissue necrosis, and stomach with symptoms of nausea, eye burns, etc.^8^. About Methylene blues’ safe exposure level, its ORAL (LDLo) for humans is 20 mg/kg bw/day. Above this dose, health effects and symptoms may occur. It can be eliminated from aqueous solutions because of its notable adsorption properties on solids^12^. Among the little cost azo dyes, Congo red (CR) is an anionic diazo-type dye that is resistant to decoloring, highly widespread, and stable in light and hard environments due to the presence of one or more chromophoric groups besides aromatic rings. Like other azo dyes, Congo red is a carcinogen dye^13,14^. This means that efforts must be applied to remove it from the industrial wastewater^15^. Regarding its toxicity value limits, it has ORAL (LDLo) 143 mg/kg for humans and ORAL (LD50) 15,200 mg/kg for animals (Rats), so, it is imperative to treat wastewater containing both dyes before draining them.

As a source of air and environment pollution and during the production activities of Portland cement, CKD, which is an undesirable by-product of solid waste, is emitted. It has the same appearance and composition as the raw materials that contribute to the manufacturing of clinker. The nano-size range of CKD can result in numerous health hazards for humans like adverse respiratory health effects, chronic obstructive lung disease, lung function impairment, pneumoconiosis, and carcinoma of the lungs^16,17^. Related to the environment, CKD has negative effects on ecosystems, vegetation, and animal health because of its content of heavy metals such as chromium, nickel, cobalt, lead, etc.^18^. It is of high value if the solid waste, especially industrial ones (CKD in the present case), can be used to purify wastewater from pollutants, thereby; one pollutant (CKD) can be applied to remove other pollutants (dyes) with relatively a little cost with high efficiency.

El Refaey and Abou-Elnasr tried to remove Imidacloprid (IMI) and oxamyl (OX) pesticides using CKD. The effects of contact time (10–360 min) and the initial concentration of each pesticide (100–2000 mg/L) were examined. The results were well satisfied with the Langmuir isotherm model (R^2^ = 0.988 and 0.999) with ultimate adsorption capacities of 142.85 and 100.00 mg/g for IMI and OX, respectively. The results revealed the suitability of using CKD as an adsorbent for IMI and OX pesticides^19^. Magdy and Altaher used the CKD for the adsorption of basic blue 69 (BB69) and Acid Red 114 (AR114) dyes by applying the batch method. They also studied the effect of various parameters like initial concentration of dye, contact time, and the mass of adsorbent as well as applying various five kinetic models. The results showed that the adsorption was chemically in nature. The adsorption degree was fast at the start of the process and affected by intraparticle diffusion and film diffusion. The equilibrium time ranged between 240 and 300 min depending on the applied dose. The ultimate adsorption magnitudes of BB69 and AR114 dyes were 2119 and 2125 mg/g, consecutively^20^. Hassan et al., applied the CKD in the removal of Murexide, Eosin yellowish, and Bromo cresol green (BCG) dyes. Among the applied isotherm models, the experimental data fitted with both Freundlich and Temkin equations for Murexide, D-R isotherm for Eosin yellowish, and Freundlich model for BCG dye. The adsorption kinetic data followed the pseudo-second-order rate. The highest removal percentages ranged between 42 and 80% at pH range 5**–**6 and the equilibrium time was reached after a maximum time of 30 min for all dyes. The adsorption was found to be physisorption in nature with less sensitivity to the temperature^21^.

The present study aims to determine the suitability of using one of the most environmentally dangerous pollutants, CKD, in the purification of colored industrial wastewater from its effluents, especially Congo red and Methylene blue dyes. The cement kiln dust powder was characterized by employing various means such as X-ray Fluorescence (XRF), X-ray diffraction (XRD), Fourier Transform Infrared Spectroscopy (FTIR), and N_2_ Brunauer–Emmett–Teller (BET) etc. These procedures were implemented besides applying various kinetic models to understand and compare the mechanism of adsorption. It is well known that, there are no previous researches that studied the uptake of both cationic and anionic dyes at the same time on CKD, also, several tests were applied for characterizing the CKD and its suitability with various analysis approaches as well as converting one harmful hazardous industrial waste to a green adsorbent for carcinogenic and toxic pollutants as it is without any modification or treatment to reduce the cost of wastewater treatment and protect the environment at the same time which provides novelty for the current research. The topic and procedures of this article agree with the global trend of waste generation and treatment^22,23^.

## Experimental procedures (materials, characterization, and methods)

### Materials

The required quantity of CKD to perform the study was imported from Alexandria Portland Cement Company (TITAN), Alexandria, Egypt. Methylene Blue (MB) dye with a formula weight of 373.90 g/mol and a maximum wavelength (λ_max_) of 663 nm and chemical formula C_16_H_18_ Cl N_2_S, and Congo Red (CR) dye with a formula weight of 696.66 g/mol and a maximum wavelength (λ_max_) equal to 498 nm and chemical formula C_32_H_22_N_6_Na_2_O_6_S_2_, were received with high purity from Merck (Sigma-Aldrich), Germany. Both sodium hydroxide (NaOH) pellets with an assay of 98–100.5% and hydrochloric acid (HCl) with an assay of ≥ 37% were ordered from Sigma-Aldrich.

### Characterization of the CKD

#### Drying of CKD powder

The CKD powder was dried in Thermo Scientific Heratherm general protocol oven from room temperature up to 80 °C and then held at that temperature for 3 h to remove any moisture content.

#### The chemical composition and the crystallographic structure of CKD

The chemical analysis of CKD was obtained by Axios max Panalytical wavelength dispersive X-ray fluorescence spectrometer, UK as shown in Table 1 which reveals the inorganic oxides that form it. The phases contained within the CKD were determined by X-ray diffraction (XRD) using a D8 Discover Bruker device within the measuring range 10–90° with a step of 0.02°.

**Table 1:** Chemical composition of cement kiln dust as obtained by XRF.

Oxide	(%)
SiO_2_	14.59
Al_2_O_3_	2.99
Fe_2_O_3_	3.60
CaO	48.24
MgO	3.32
SO_3_	4.42
Na_2_O	2.59
K_2_O	6.55
Cl	8.58
LOI	7.58

### Determination of point of zero charge (PZC)

PZC is determined to investigate the values of pH at which the surface charges of CKD become negative, positive, or neutral in the aqueous media. For the determination of the PZC of CKD, the pH drift method was applied through the next steps. 50 mL of 0.1 M KNO_3_ solution was added to a set of 100 mL conical flasks. A range of 2–12 pH was achieved by adding drops of HNO_3_ and NaOH (0.1 mol/L) to the solution. A dose of 2 g/L of CKD was added to the flasks and stirred for 24 h at room temperature and the final pH values were determined. The initial pH versus ∆pH (pH _initial_−pH _final_) was represented to specify the PZC of CKD. The pH was measured using the Jenway 3510 pH meter, UK.

#### FTIR spectrum

Fourier transformation infrared (FTIR) spectra for the CKD powder were obtained using a Nicolet 380 FTIR spectrometer, USA. The powder was mixed with KBr and pressed to form pellets. The measurement was performed at room temperature within the range of 4000–400/cm with a resolution of ± 1/cm.

#### Brunauer–Emmett–Teller (BET) test

This test was carried out to obtain and analyze the N_2_ gas adsorption–desorption isotherms to determine the specific surface area, total pore volume, and the mean pore diameter. The test was performed by a fully automated Micromeritics ASAP 2020 surface area analyzer, USA, using the N_2_ adsorption–desorption method to obtain the BET isotherms at 298 K room temperature. The test duration was 4 h using 0.2806 g of the sample.

#### Zeta potential and particle size distribution (PSD) analysis of CKD

Zeta potential is a measure of the effective electric charges on the particle surface. The magnitude of zeta potential delivers information about particle stability with particles of higher zeta potential magnitude showing increased stability due to a larger electrostatic attraction among particles. On the other side, PSD is a measurement designed to determine and report information about the size and range of sizes of distributed CKD powder from sub-nanometers to several micrometers in diameter using the performance of dynamic light scattering. Both the Zeta potential and particle size distribution were determined at room temperature by using Zetasizer, Ver. 7.10, Malvern Instruments Ltd, UK.

#### Scanning electron microscopy (SEM)

The morphological features and properties of the CKD adsorbent before and after performing the adsorption of dyes were examined using the SEM technique. The apparatus used was JEOL- JSM-IT200 Series. The CKD powder was coated with a thin coating layer of gold in a JEOL ion sputtering device (JFC-1100E) before the examination. The SEM images were captured at various magnification scales before and after the sorption process for distinction purposes.

### Methods

#### Preparing the solutions of model dyes

The industrial wastewater required to determine the adsorption behavior of CKD was experimentally simulated by preparing stock solutions of 1000 ppm of MB and CR dyes by dissolving 1 g of such dyes in one liter of water. These stock solutions were diluted with distilled water following serial dilution to obtain the calibration curves and to prepare the required experimental solutions with various concentrations of 25, 50, 100, 150, and 250 ppm.

#### Adsorption test

Adsorption experiments were executed by applying the batch adsorption method for both MB and CR dyes. Factors such as initial dye concentration, contact time, CKD dose, and the effect of temperature were studied and evaluated. All tests were carried out in 250 ml volumetric flasks and stirred on a shaker at 300 rpm. Centrifuging at 4000 rpm was carried out for the solutions to separate the dye from the adsorbent. The final concentrations of dyes were detected by using a Jenway 7415 scanning UV/visible spectrometer cloud connectivity, England, with wavelength accuracy of ± 2 nm. The amount of the adsorbed dyes at the equilibrium $Q_{{\varvec{e}}}$ (mg/g) as well as the percentage of the dye removal efficiency (R%) from the aqueous solutions were determined using Eqs. (1) and (2), respectively^24^.

1 $$ Q_{e} = \frac{{(C_{i} - C_{e) } }}{m} \times V $$

2 $$ {\text{R}}\% = \frac{{(C_{i} - C_{e)} }}{{C_{i} }} \times 100 $$

where $$C_{i}$$, $$C_{e}$$,$$ m$$, and $$\user2{ }V$$ are the initial concentration of the dye (ppm), the dye concentration at equilibrium (ppm), the initial CKD dose (g), and the solution volume (L) which was fixed (0.1 L) for all tests, respectively.

#### Impact of contact time and kinetic modeling

The amount of adsorption was measured against time to understand the adsorption kinetics behind the CR and MB dyes removal processes. The tests were performed by mixing 0.25 g of CKD powder with 100 ppm of MB dye solutions and 0.25 g of the CKD with 250 ppm of CR dye solutions ‘respectively’ at room temperature and constant pH for time intervals 20, 40, 60, 80, 100, 120, 140, 160 and 180 min.

For kinetic modeling, linear pseudo-first-order, linear pseudo-second-order, and intra-particle diffusion models were utilized to understand the adsorption mechanism of CR and MB dyes on CKD powder. The applied kinetic modeling equations can be found in detail in the references^25–27^.

#### Effect of pH

Generally, the pH of the solution impacts the adsorbent's surface charges. In order to find the ideal conditions for dye removal, the impact of pH on the adsorption mechanism should be discussed. This can be achieved by performing experiments with various initial pH that have been adjusted to be 2, 4, 6, 8, 10, and 12 with 0.1 M HCl and 0.1 M NaOH. Tests were carried out by mixing 0.50 g of the CKD with 50 ppm of MB dye solution and 0.5 g of CKD with 100 ppm of CR dye solution at room temperature and a constant contact time of 120 min.

#### Effect of initial CKD mass and isotherm models

Various initial masses of CKD precisely 0.10, 0.25, 0.50, 0.75, 1.00, 1.25, 1.50, 1.75, and 2.00 g were used with MB and CR dyes with concentrations of 150 and 250 ppm at room temperature, constant pH, and 120 min contact time conditions. The obtained data have been used to discuss both Langmuir and Freundlich isotherm adsorption models^25–27^ which are essential to understand the adsorption behavior of the dyes on the solid adsorbent.

Langmuir is predicated on the concept that the dissolved dye is adsorbed as a monolayer form at predetermined homogeneous sites across the adsorbent where all sites are similar and energetically equivalent^28^. Langmuir’s linear form is mathematically formulated as expressed in Eq. (3).

3 $$ \frac{{C_{e} }}{{Q_{e} }} = \frac{1}{{b Q_{max} }} + \frac{{C_{e} }}{{Q_{max} }} $$

where $$b$$ and $$Q_{max}$$ are Langmuir constant correlated with the energy of the process of the adsorption (L/mg) and the maximum quantity of dye at complete monolayer coverage per unit mass of CKD (mg/g), respectively. The values of $$b$$ and $$ Q_{max}$$ can be figured out from the intercept and slope of the $$\frac{{C_{e} }}{{Q_{e} }}$$ versus $$C_{e}$$ plot.

The equilibrium Langmuir adsorption separation constant (R_L_), which is the main feature of the model, can be determined from Eq. (4).

4 $$ {\text{R}}_{{\text{L}}} = \frac{1}{{\left( {1 + bC_{i} } \right)}} $$

where $C_{i}$ is the initial concentration of the dye. Values of R_L_ can be used to specify the type of adsorption, where when its value = 0 this means that the adsorption nature is irreversible and adsorbate cannot be removed. When the value of R_L_ is between 0 and 1 this indicates that the process of adsorption is favorable. When R_L_ becomes larger than 1 this means that the process is unfavorable. Finally, when R_L_ = 1 this denotes a linear adsorption process^25,29^.

The non-linear expression of Langmuir isotherm model is indicated by Eq. (5)

5 $$ {\text{q}}_{{\text{e}}} { = }\frac{{{\text{Q}}_{{{\text{max}}}} {\text{ b C}}_{{\text{e}}} }}{{{\text{1 + b C}}_{{\text{e}}} { }}} $$

Another applied model is Freundlich adsorption which can be used to describe the non-ideal heterogeneous multilayer adsorption with linear form. The model assumes that the adsorption exists on a heterogeneous surface and involves unequally numerous sites of various adsorption energies^30^. The model is mathematically formulated linearly as in Eq. (6).

6 $$ \ln Q_{e} = \ln K_{f} + {\raise0.7ex\hbox{$1$} \!\mathord{\left/ {\vphantom {1 n}}\right.\kern-0pt} \!\lower0.7ex\hbox{$n$}}{\text{ ln}}C_{e} $$

where $$K_{f}$$ and $$ n$$ are Freundlich constants that reveal the adsorption capacity (mg g^-1^), and the adsorption intensity, respectively. Values of $$K_{f}$$ and $$ n$$ can be figured out using the intercept and the slope of $$\ln Q_{e}$$ versus $${\text{ ln}}C_{e}$$ plot.

The nonlinear form of the Freundlich isotherm model is mathematically formulated as in Eq. (7) ^7^.

7 $$ Q_{e} = K_{f} C_{e}^{{{\raise0.7ex\hbox{$1$} \!\mathord{\left/ {\vphantom {1 n}}\right.\kern-0pt} \!\lower0.7ex\hbox{$n$}}}} $$

#### Effect of temperature and thermodynamic parameters

To better comprehend how the temperature affects the uptake of both MB and CR dyes using CKD adsorbent, tests were carried out by mixing 0.25 and 0.75 g of the CKD with 100 and 250 ppm of MB dye solutions ‘respectively’ and 0.25 and 0.50 g of the CKD with 50 and 250 ppm of CR dye solutions ‘respectively’ at various 298, 308, 318, and 328 K temperatures at constant pH with 300 rpm, and 120 min of contact time.

The mathematical formulations of the standard thermodynamic parameters such as the change in the Gibbs free energy ($\Delta {\text{G}}^{^\circ }$), enthalpy ($\Delta {\text{H}}^{^\circ }$), and entropy change ($\Delta {\text{S}}^{^\circ }$) can be found in detail in the references^11,24^.

## Results and discussion

### Characterization of the starting CKD

#### Point of zero charge (PZC) of CKD

**Figure 1:**
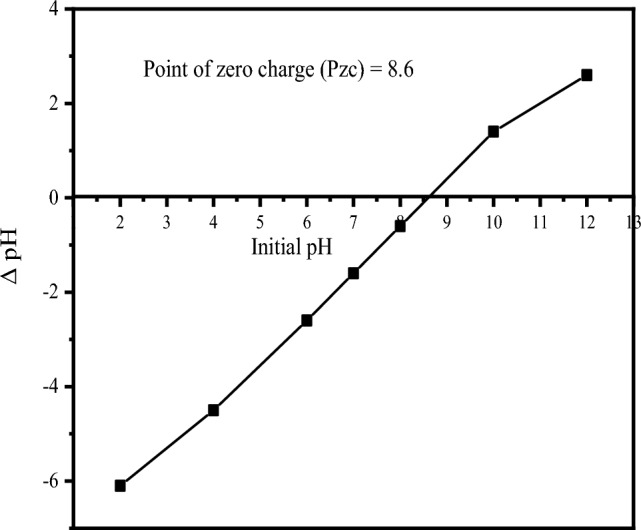
Determining the point of zero charge of CKD.

Where the point of zero charge affects the adsorption process under certain pH conditions, Fig. 1 shows the PZC of CKD adsorbent that results from the intersection of ∆pH with the initial pH following the drift method^31^. The PZC value is found to be 8.6. Below this pH value, the surface of CKD is positively charged and above it, its surface is negatively charged, while particularly at this value the absorbent's surface becomes a net electrically neutral. Subsequently, the favorable conditions for the uptake of cationic dye, i.e., MB, are achieved when the pH of the medium becomes higher than 8.6 while the encouraging conditions for the adsorption of CR anionic dye are less than this value because of the attraction that arises between the surfaces of both the adsorbent and the adsorbate in both cases. These facts are in agreement with previous researches^11,15,31^. This value, i.e., 8.6, was found to agree with previous studies of the point of zero charge of CKD^32^.

#### XRD analysis results

**Figure 2:**
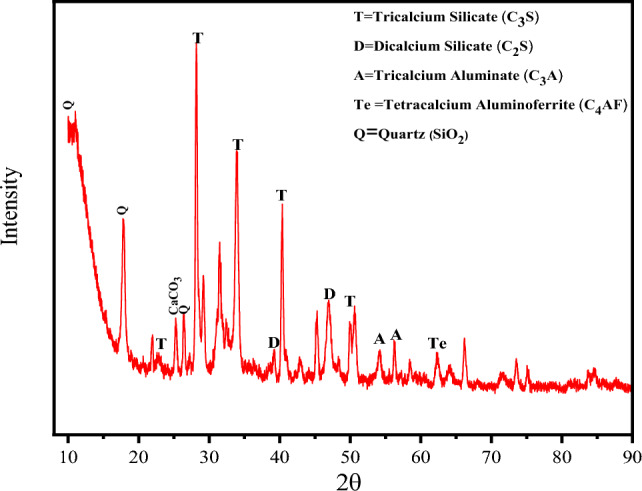
X-ray diffraction spectrum of CKD.

The XRD crystallography of the cement kiln dust powder, as revealed in Fig. 2, proves the presence of CaO peaks in the extent of 31–64° while the peaks of SiO_2_ are in the range 17.5–86°. The phases found in the XRD are Di and Tricalcium Silicate, Tricalcium Aluminate, Tetracalcium Aluminoferrite, and Quartz as confirmed from both the previous studies^33–38^ and also the XRF results.

#### BET test

**Figure 3:**
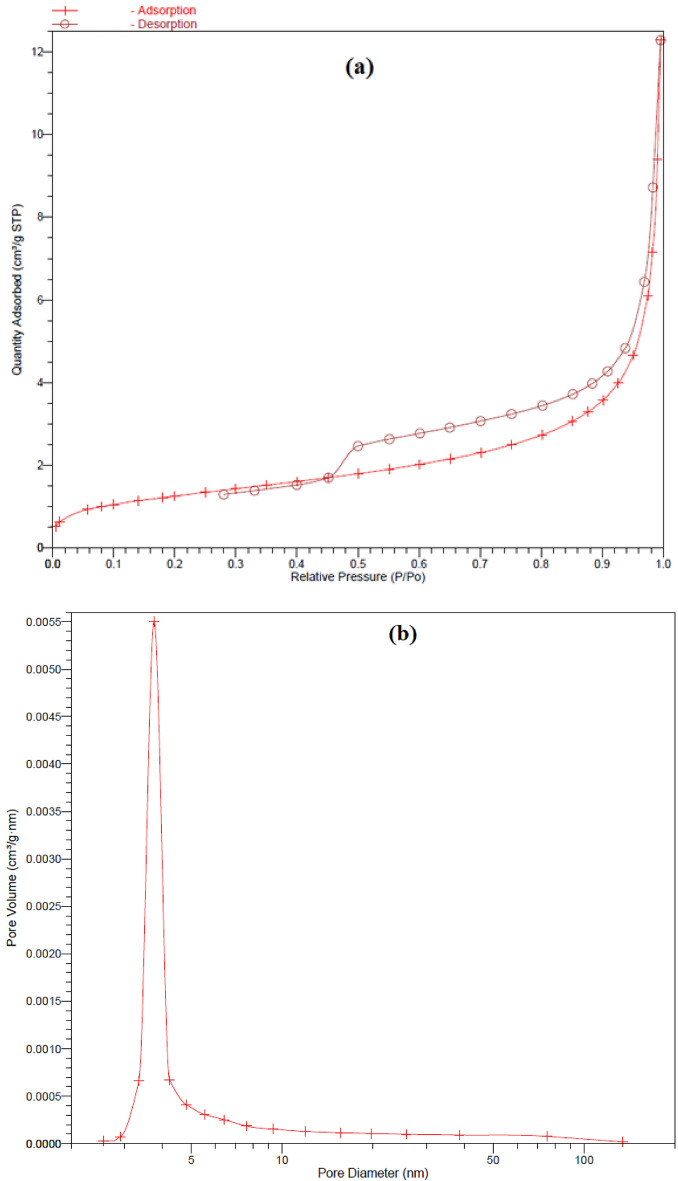
(**a**) N_2_ adsorption–desorption isotherms and (**b**) pore size distribution of CKD.

Figure 3a represents the standard N_2_-BET isotherm, that meets an IV Type isotherm as per IUPAC classification, which reveals the rising of the volume of the adsorbed N_2_ gas with increasing the relative pressure (P/P_o_) for CKD powder indicating initially that the adsorbent is mesoporous. The Barrett–Joyner–Halenda (BJH) pore size distribution curve of CKD, Fig. 3b, reveals that the major particle diameter is in the extent of 3 − 5 nm. From the test, it can be found that values of the BETs’ CKD surface area, BJH adsorption cumulative volume of pores, as well the mean pore diameter at BJH adsorption are 4.5056 m^2^/g, 0.018085 cm^3^/g, and 21.2432 nm, respectively. The results also revealed that the average particle size in the nanoscale is 1331.6669 nm. These BET test results are key parameters that impact the adsorption performance or adsorption capacity.

#### Zeta potential and PSD of CKD

**Figure 4:**
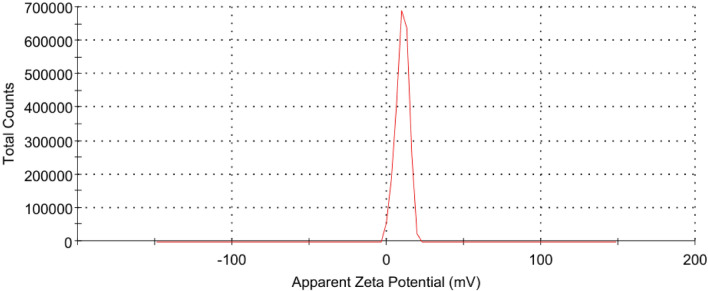
Zeta potential distribution of CKD suspension.

**Figure 5:**
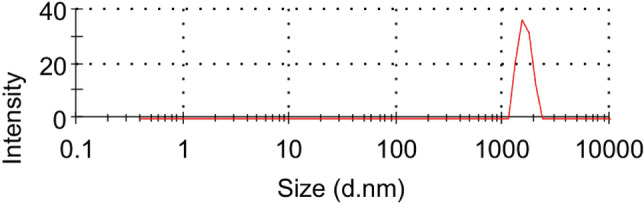
Zeta size distribution by intensity of CKD particles.

Figure 4 shows the zeta potential distribution of CKD which is a positive value (+ 9.94 millivolts) confirming the presence of stable CKD particles in the solution. Figure 5 reveals the size distribution histogram by intensity of CKD suspensions. The curve shows one peak with a narrow size distribution in the histogram which may be related to the existence of one type of size. The particle size of CKD was found to be about 1399 nm (1.39 μm). This size confirms the average particle size obtained from the BET test in the previous section. Also, this finding reveals that the particle size of CKD is highly monodispersed at about 738 nm indicating that they have the same size. The highly monodispersed particles of CKD in the solution in addition to the high surface area of the particles can help in CKD particles’ spreading during the adsorption process leading to their high adsorption capacity.

#### FTIR spectra of CKD and CKD loaded with MB & CR dyes

**Figure 6:**
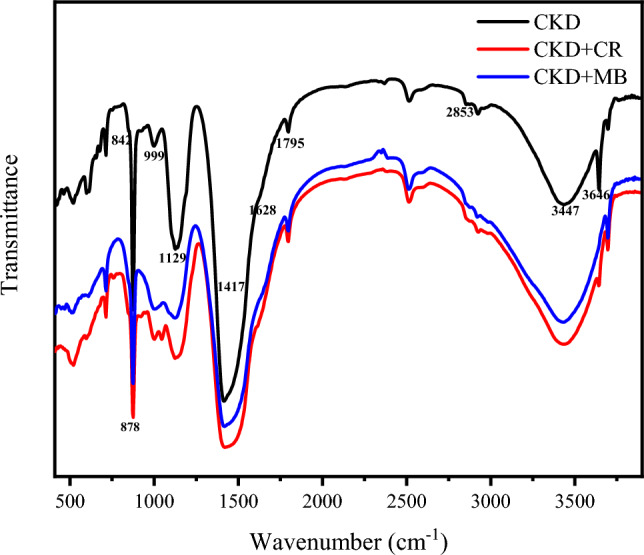
FTIR spectra of CKD, CKD loaded with MB and CR dyes.

This test was performed to detail the structural units embedded within the CKD powder as can be revealed in Fig. 6. The peaks around 878 and 842/cm, and 1129/cm are attributed to the bending vibrations of Si–O bonds in both C_2_S and C_3_S phases, respectively. The peak observed at 999/cm indicates the Si–O–Si bond. The peak around 1628/cm is ascribed to Si–O–Si asymmetric vibration that may also overlap with the aromatic C=C peak. The small peak at ≈ 1795 is attributed to O–H (hydroxyl). The small shoulder observed at 2853/cm may be related to C–H stretching in alkanes. The peak at 3646 is due to the O–H stretch of calcium hydroxide mineral^19,33,34^. The peak at ≈ 3474/cm is due to O–H stretching vibrations of the adsorbed water on cement kiln dust phases. The band at ≈ 1417/cm is assigned to the asymmetric stretching of carbonate CO_3_^2-^ molecules that result from the reaction of calcium hydroxide with the atmospheric air^35^. The shift of CKD functional groups’ bands, and decreasing the bands' intensity like bands at 1417 and 878/cm, also the appearance of the characteristics bands and shoulders related to the dyes, like shoulders at 1610/cm for CR and 1599 for MB^12^, on the spectrum of CKD, as well as other minor changes in the spectra of the CKD loaded with such dyes confirm the reaction and adsorption of both MB and CR dyes molecules on CKD particles surface. Related to the CR dye, the more shifts of peaks such as the peak at 1416–1428/cm as well as the splitting of the peak at 999 related to the Si–O–Si bond^36^ and other shifts confirm the formed hydrogen bond between the CKD particles and the absorbed dye as will be discussed in the proposed mechanism of adsorption.

#### SEM micrographs before and after dyes adsorption

**Figure 7:**
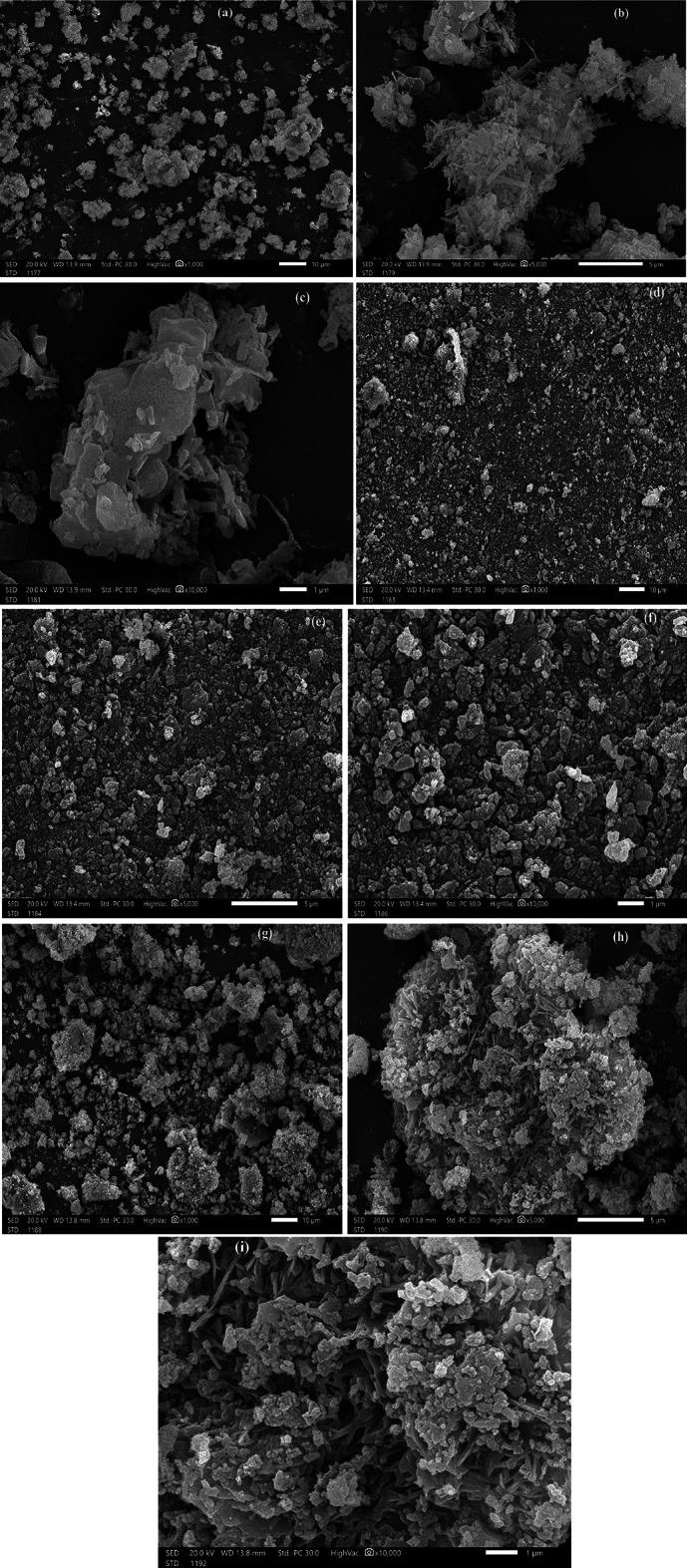
SEM illustrations of pure cement kiln dust (CKD) (**a**–**c**), (**d**–**f**) for CKD after adsorption of MB, and (**g**–**i**) after adsorption of CR, respectively.

SEM photomicrographs of the native CKD (Fig. 7a–c) reveal the presence of holes and pores in its layers and the structure is strip-like, especially when the photo is captured at high magnification. This is typically matched with previous studies that used and characterized the CKD^19^. The shape of the CKD surface changed after the adsorption occurrence of MB (Fig. 7d–f) and CR (Fig. 7g–i) dyes demonstrating the existence of new formations and the surface (strips) of CKD is wrapped with these molecules. The indicated variation in the CKD photomicrographs before and after adsorption (in general) and between the CKD loaded with MB and CR affirms the physical mechanism of adsorption of both dyes in the forms of monolayers and multi-layers on the CKD surface.

### Effects of various parameters on the removal of MB and CR dyes by CKD

#### Effect of CKD mass

**Figure 8:**
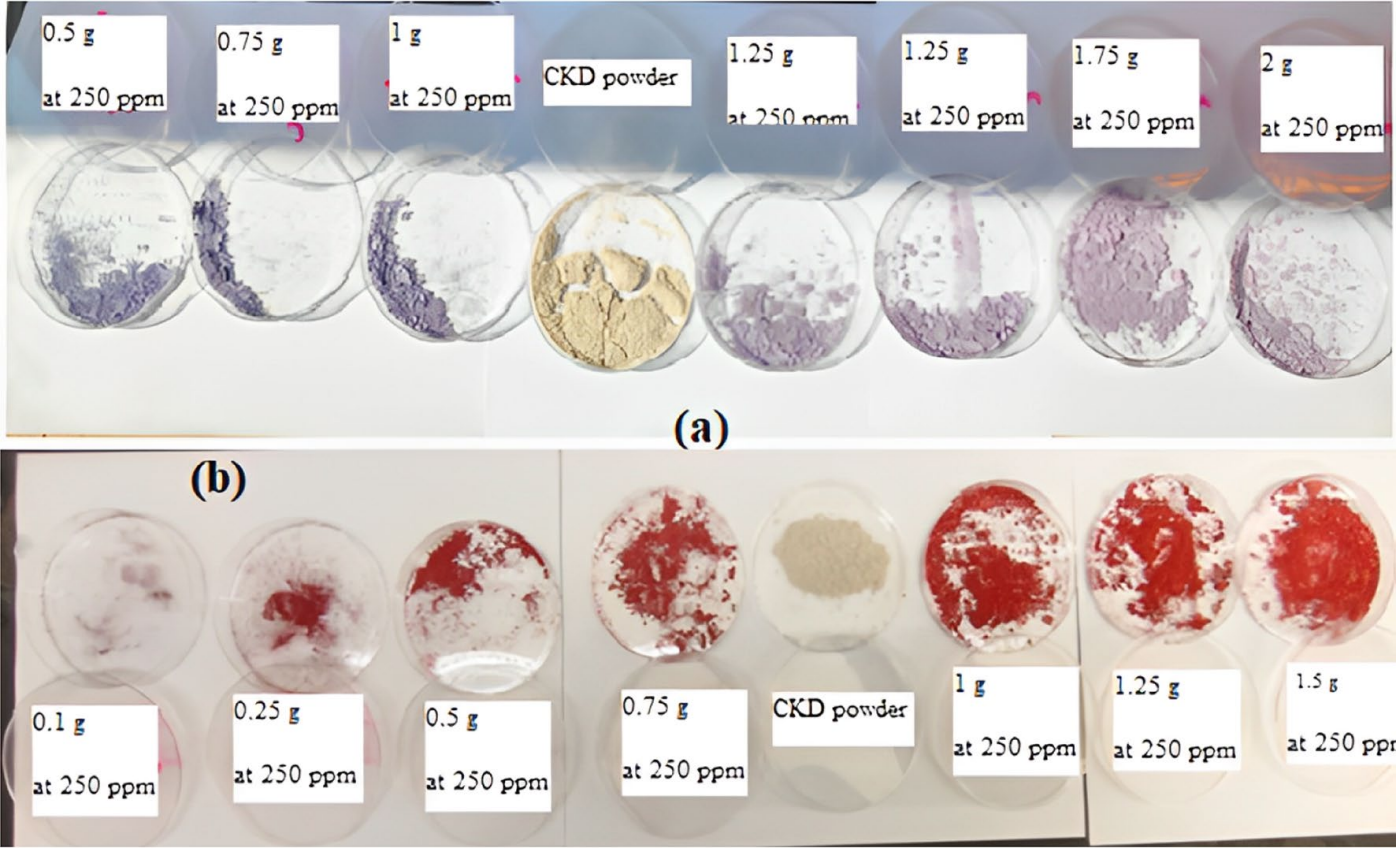
Different masses of CKD powder loaded with (**a**) MB and (**b**) CR dyes.

**Figure 9:**
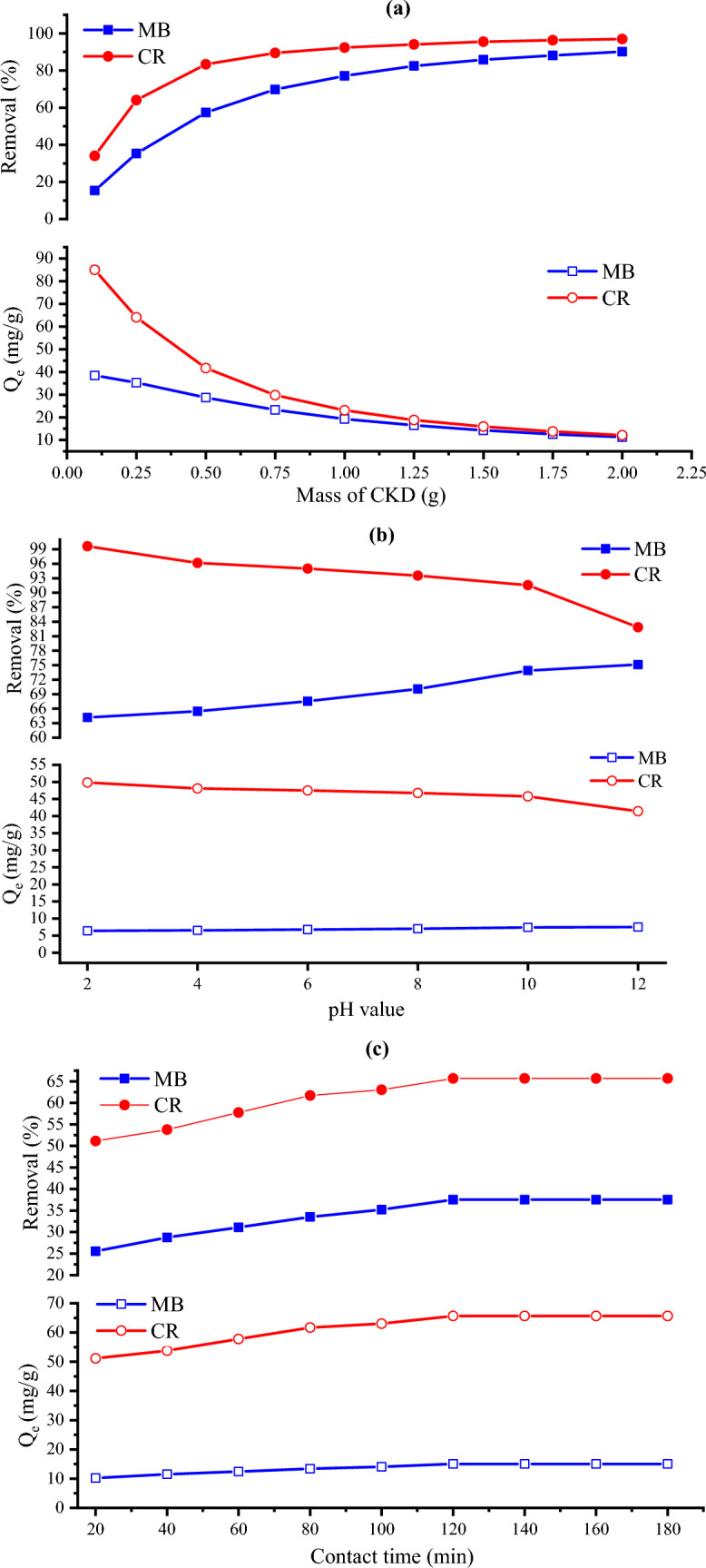

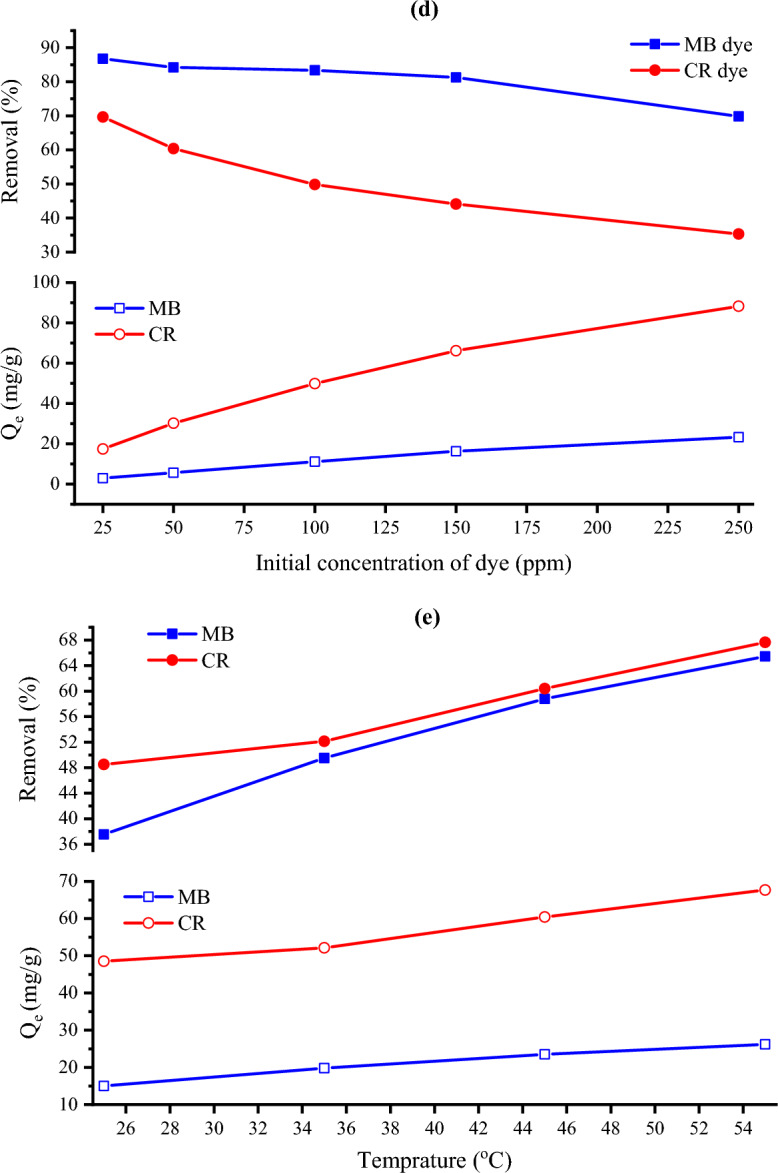
The effect of (**a**) CKD dosage, (**b**) pH value, (**c**) contact time, (**d**) initial dye concentration, and (**e**) temperature on the removal (%) and the amount of equilibrium adsorption (Q_e_) of MB and CR dyes.

The effect of various initial masses of CKD on the elimination of both MB and CR dyes was studied at 300 rpm agitation speed, 12.1 pH, 120 min contact time, and (25 °C) room temperature conditions. The used various masses of CKD powder loaded with MB and CR dyes are shown in Fig. 8a and b. The removal trend in addition to the amount of equilibrium adsorption for such dyes is depicted in Fig. 9a which shows inverse behavior between both. From the figure, it can be seen that the removal of MB and CR dyes increases with a further increase in the CKD dose from 0.1 to 2 g, and the optimum doses that achieved the highest adsorption for both dyes and after them plateau phenomenon occurs are 1.75 g for MB and 1.25 for CR. This removal increase is ascribed to the high amount of CKD with low particles’, and consequently pores’, size that exhibit high surface area and hence more dye adsorption sites.

#### Influence of solutions’ pH

The solutions’ pH is another factor that affects the dye sorption by influencing the CKD functional groups, and surface charges, in addition to the dye molecules' ionization action. The effect of altering pH from 2 to 12 is shown in Fig. 9b using 0.5 g of CKD, 300 rpm agitation speed, and 120 min contact time at room temperature (25 °C) conditions. The figure shows that the adsorption of MB dye drastically increases with increasing the pH value. The maximum uptake for the dye was found at pH 12 value. This is attributed to the cationic nature of the dye which is loaded with positive charges that make the attraction to the CKD adsorbent surface (which is highly negatively charged at higher pH values) increases with rising pH value. The inverse of this situation can be used in interpreting decreasing the removal of CR dye with increasing the pH value. As seen in Fig. 9b, rising the pH value leads to increasing the negative charges of the medium leading to more repulsion between the dye and adsorbent surface, besides the anionic nature of CR dye which decreases the overall attraction of the CR dye molecules. The pH 2 value witnesses the maximum attraction between the CR dye and the CKD surface providing the maximum adsorption. This fact is supported by the PZC of CKD, which is 8.6, below which the surface is positively charged increasing the attraction between the anionic CR dye and the powder surface giving the highest adsorption at pH 2.

#### The influence of contact time

The contact time is defined as the time needed to balance the adsorption/desorption or the time wanted to reach the saturation equilibrium by the substrate^31^. The influence of varying the contact time from 20 to 180 min on the adsorption process performance was studied and represented in Fig. 9c. The test was carried out at 100 ppm dye concentration, 0.25 g of the adsorbent, pH = 12.2, 300 rpm, room temperature (25 °C) conditions for MB dye, and 250 ppm dye concentration, 0.25 g of the adsorbent, pH 12.2, 300 rpm, room temperature (25 °C) conditions for CR dye, respectively. The graphical representation of the contact time effect shows that the dye removal effectively increases at first 100 min under the applied operating conditions then the optimum contact time was achieved at 120 min. After this contact time, the adsorption of the dye was not affected by the time showing a balanced state. The fast adsorption at the first 120 min is ascribed to the available large number of active vacant adsorption binding sites before the equilibrium occurrence. After that time, the adsorbent sites are saturated with dye molecules, decreasing more adsorption. Finally, dynamic equilibrium occurs with a maximum value and at this time the rate of adsorption becomes equal to the rate of desorption^15^. This indicates that the adsorption process is contact time dependent. The importance of studying this factor comes from its influence on the adsorption process where a sufficient contact time allows more chances for the purification of the contaminated water permitting more adsorption for the dyes. The steady-state adsorption time in the present case was reached after a time equal to or less than the equilibrium time that has been reached by other various low-cost adsorbents for the uptake of MB dye^5,8,11^, or CR dye^15,39^.

#### The influence of initial dye concentrations

This factor means for the influence of rising the dye concentration on the adsorbed amount of dye on CKD powder. The effect of changing the dye concentration from 25 to 250 ppm was studied at room temperature (25 °C), 12.2 pH, 300 rpm, 120 min contact time conditions, and 0.75 g initial mass of CKD for MB and 0.1 g for CR dye, respectively. Figure 9d shows the effect of increasing the dye concentration on both the removal process and the amount of the equilibrium absorbed dye, which reveals a decreasing trend and an inverse relation between the removal process and the concentration of the adsorbate. This decrease in such dyes’ removal is attributed to the competition of the adsorbate molecules and the saturation of the adsorption active sites of the adsorbent with increasing the concentration of the dye from 25 to 250 ppm. Another possible reason for decreasing the removal of dye is reducing the mass transfer between the solution and the adsorbent at high concentrations of dye^24^.

#### Effect of varying the temperature on the dye removal

**Figure 10:**
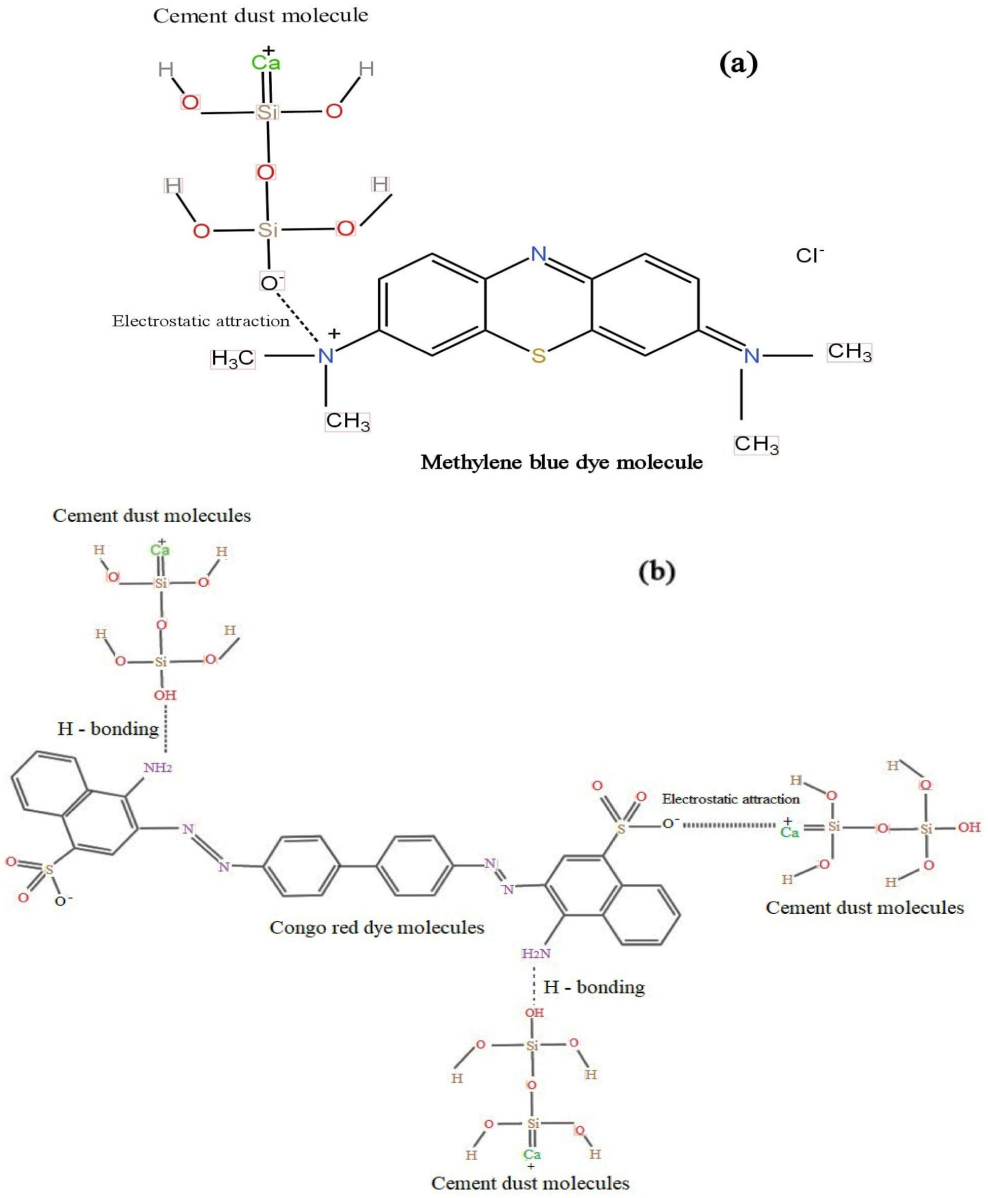
Schematic representation of the adsorption mechanism of (**a**) MB and (**b**) CR dyes on CKD surface.

The impact of varying the solution temperature from 25 to 55 °C and fixing the pH at 12.2, the centrifuging speed at 300 rpm, the contact time at 120 min, and the initial mass of CKD for MB at 0.25 g and 0.1 g for CR dye ‘respectively’ on the adsorption of such dyes as well as $(Q_{e}$) is represented in Fig. 9e. The data show that the dye removal from the solution increases with rising the temperature for both dyes which means that the temperature has a positive effect on the dye uptake. This can be attributed to the increment in the kinetic motion of the adsorbate molecules with rising the temperature that increases their mobility, by adding power to them, facilitating their spread rate across the solution towards the external layer and the inner pores of CKD that aid in the creation of surface monolayer’s^5,26,40^. Another possible reason for this behavior is the fact which is proposed by Singh et al. that increasing the number of binding sites of the dye molecules on the adsorbent surface with rising temperature increases the dyes’ uptake on the adsorbent covering^41^. These results support the fact that the process is chemisorption (as will be confirmed from the thermodynamics analysis); hence, more removal can be acquired with rising the temperature. Comprehensively, it can be seen that the mechanism of the uptake of such dyes is found to be highly pH-dependent. At high pH (above Pzc), which witnesses the maximum adsorption of cationic MB dye, electrostatic attraction occurs between the negatively charged surface of CKD particles and the positively charged groups of the MB dye^42–44^. On the other side, CR dye adsorption can be accomplished via a combination of two mechanisms: firstly, electrostatic attraction between negatively charged CR molecules and positively charged CKD particles (lower than PZC of CKD); and secondly, the possible formation of two hydrogen bonds among the amino groups of CR with hydroxyl groups of CKD particles^30,45–47^. This mechanism is represented in Fig. 10. This is why the CR dye is notably absorbed by the CKD more than the MB dye.

#### Study of the adsorption kinetics

**Figure 11:**
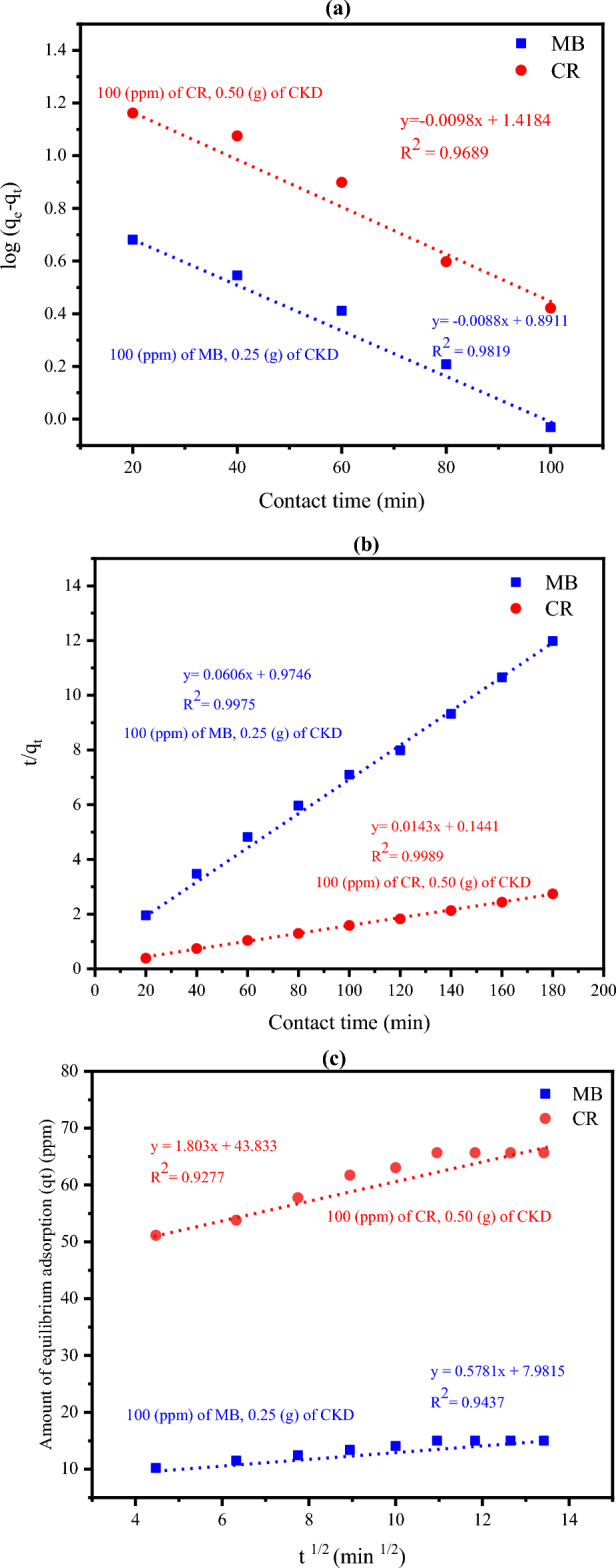
(**a**) Pseudo-first-order (**b**) pseudo-second-order, and (**c**) intra-particle-diffusion kinetic models plots for the adsorption of MB and CR dyes on the surface of CKD particles.

The linear forms of the three kinetic models namely: pseudo-first-order, pseudo-second-order; and the model of intra-particle diffusion were applied to identify the adsorption mechanism of MB and CR dyes on CKD powder as represented in Fig. 11a, b, and c. The kinetic parameters applying the three various kinetic models are tabulated in Table 2. The data in both Fig. 11 and Table 2 reveal that the kinetic data are best fitted and in excellent agreement with the pseudo–second–order kinetic model at all times along the whole adsorption process rather than the other studied models. This is in agreement with a previous study that used the cement nano-composite in the adsorption of both MB and CR dyes^48^. The regression coefficient (R^2^) for the model is higher than 0.997, very near to the unit, and it is not only dependent on the initial concentration of the dye^49^. The uptake mechanism of such dyes on the exterior of CKD passes through numerous various stages. Firstly, the dye molecules move towards the aqueous thin layer that surrounds the CKD, diffusing it in what is known as external diffusion. After that, the adsorbate particles penetrate the pores found on the CKD surface (intraparticle diffusion). Finally, the adsorption of dye molecules occurs on the internal cavities of CKD particles^19,20^.

**Table 2:** The kinetic parameters for the removal of MB and CR dyes by using the CKD applying different kinetic models.

The kinetic model	The parameter	The value related to the dye	
		MB	CR
Pseudo-first-order	$K_{1}$(/min)	0.0203	0.0225
	$q_{e1}$(mg/g)	7.781	4.130
	R^2^	0.9819	0.9689
Pseudo-second-order	$K_{2}$(g/mg min)	0.004	0.001
	$q_{e2}$(mg/g)	16.515	69.794
	R^2^	0.9975	0.9988
Intra-particle diffusion	$k_{id}$(mg/g min^1/2^)	0.578	1.803
	$C$	7.981	43.832
	R^2^	0.9437	0.9277

#### Study of the adsorption thermodynamics

**Figure 12:**
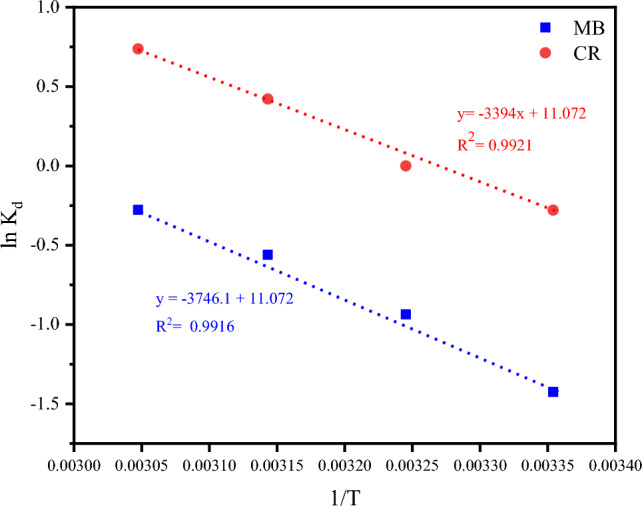
The plot of ln K_d_ versus 1/T for the adsorption of MB and CR dyes on CKD particles.

As stated earlier, the values of standard $$\Delta {\text{G}}^{^\circ }$$, $$\Delta {\text{H}}^{^\circ }$$, and $$\Delta {\text{S}}^{^\circ }$$ were computed from the slope and intercept of $$\ln K_{d}$$ versus $${\raise0.7ex\hbox{$1$} \!\mathord{\left/ {\vphantom {1 {\text{T}}}}\right.\kern-0pt} \!\lower0.7ex\hbox{${\text{T}}$}}$$ plot (i.e., the change of equilibrium constant with the inverse of temperature) as represented in Fig. 12. Table 3 reveals values of the thermodynamic parameters for both dyes. From the table, one can confirm the endothermic nature of the adsorption process of such dyes on CKD particles due to the positive values of ΔH. Additionally, the values of ΔH of the present study (28.305 and 31.242 kJ/mol) which lie in the range (1–60 kJ/mol) confirm the physisorption nature of the adsorption process^50^. Concerning the ΔS° values, their positive values, as in the present case, show a good attraction of dye molecules on the CKD surfaces, and their motion is not restricted and the randomness is growing at the solid/liquid interface with increasing the temperature in the time of the adsorption process^11,13^.

**Table 3:** Thermodynamic parameters of MB and CR dyes adsorption.

Parameter	ΔG° (J/mol)				ΔH° (kJ/mol)	ΔS° (J/K mol)
Temperature (K)	298	308	318	328		
MB dye	3447	2514	1582	650	31.242	0.0930
CR dye	779	− 143	− 1066	− 1990	28.305	0.0923

#### The adsorption isotherms models

**Figure 13:**
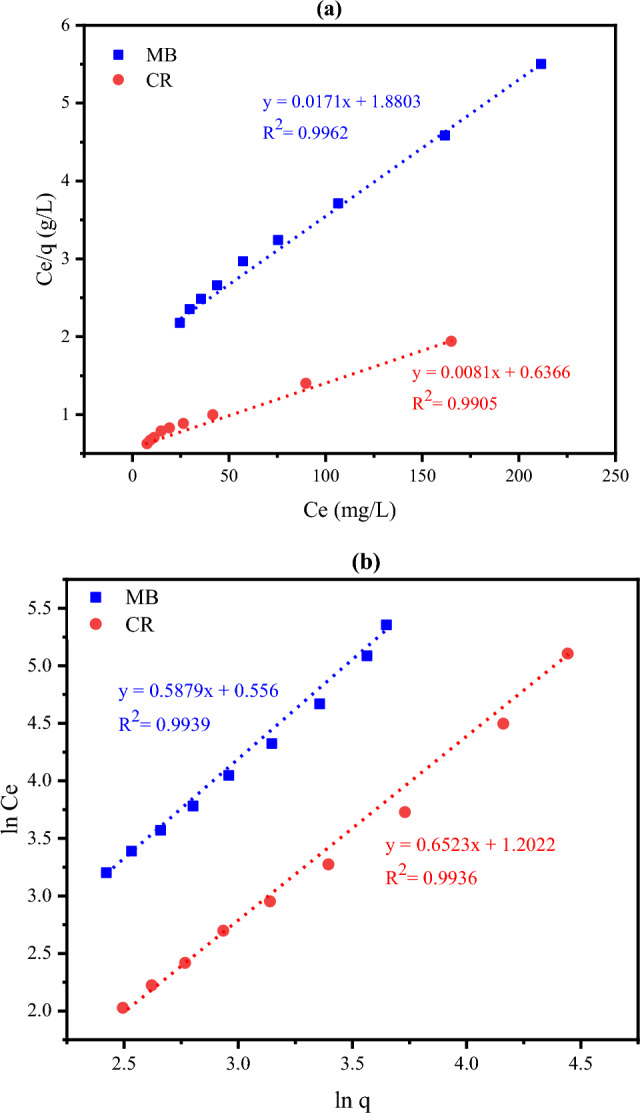
Linear (**a**) Langmuir and (**b**) Freundlich isotherms’ parameters for MB and CR dyes adsorption process on CKD surface.

**Figure 14:**
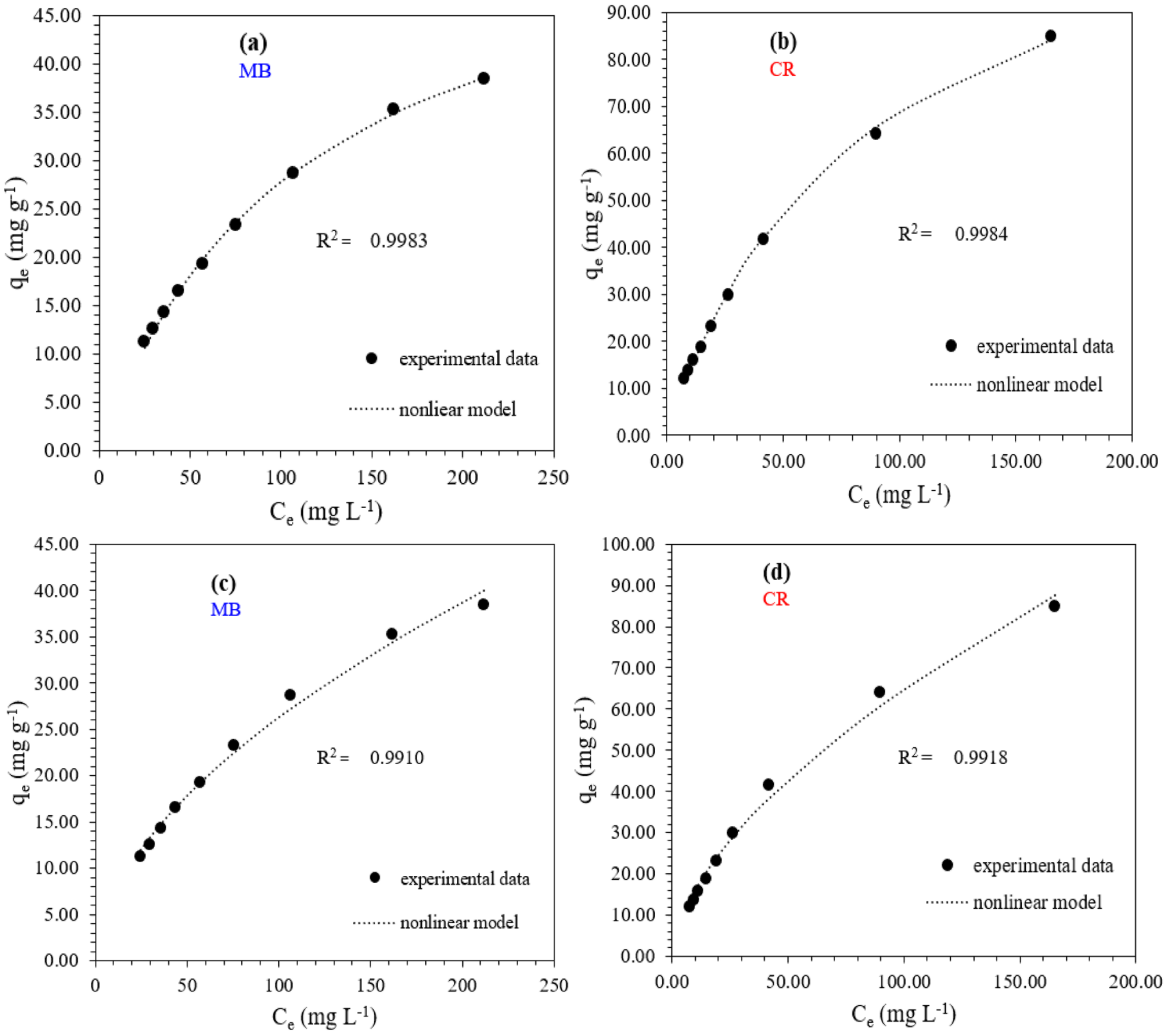
Nonlinear fittings of Langmuir model for (**a**) MB and (**b**) CR dyes and Freundlich model for (**c**) MB and (**d**) CR dyes, respectively.

The two isotherms’ models of Langmuir and Freundlich were applied to investigate the correlation between the quantity of the adsorbed dye and its concentration in the solution at constant temperature when the equilibrium state is achieved and to determine the adsorption mechanism (the interaction) at the adsorbent-adsorbate interface^43^. The Langmuir isotherm theory supposes that the adsorption occurs in the form of monolayer coverage of the adsorbate over a homogenous adsorbent surface on homogeneous sites with equal energies. On the other hand, the Freundlich empirical equation assumes that the adsorbent surface has available sites with various strong binding energies where the uptake of the adsorbate can take place and the quantity of the absorbed material is in direct relation with its concentration. Both Fig. 13 and Table 4 reflect the adsorption isotherm parameters for MB and CR dyes applying both models revealing the subsequent results. First, it was found that the dimensionless separation factor R_L_ values are 0.305 and 0.239, which lie between 0 and 1, indicating that the adsorption of such dyes on the CKD surface is favorable under the experimental conditions, as confirmed by the Study of the adsorption thermodynamics section. Secondly, R^2^ values of correlation coefficients were used to determine the applicability of such isotherm models to the experimental data of the adsorption study or the suitability of the adsorbent to each studied model. The very relative values of the regression coefficient (R^2^) for both models (> 0.9905) reveal that the adsorption of MB and CR dyes achieves both homogeneous mono-layer, one molecule thickness, and heterogeneous multilayer coverage on the outer surface of equal and uneven adsorption energies active sites of CKD particles at the same time, and both Langmuir and Freundlich isotherms are in a better fit to the experimental adsorption data^13^. Thirdly, the adsorption capacities, i.e., the amount of dye absorbed per gram of CKD, were found to be 58.43 and 123.42 mg/g for MB and CR dyes, respectively. These values of adsorption capacities are nearly the same as multi-dye nanoparticle adsorbents^51^ or higher than the optimal adsorption capacities for some other high-cost treated adsorbents as seen in Table 5 which add value to the little-cost CKD adsorbent used in the present case. On the other side, the non-linear mode of both Langmuir and Freundlich isotherm models was applied for both dyes to find out the best fit for the experimental equilibrium data and to avoid the inherent bias and errors that may arise from the linearization. The nonlinear findings were compared with the linear ones as seen in Table 4. The representation of the deviations and the fittings of the experimental data applying both models, which are indicated in Fig. 14, besides the non-linear R^2^ values for both models tabulated in Table 4 support the aforementioned linear findings that both Langmuir and Freundlich isotherm models match the experimental equilibrium adsorption linear data well. Finally, the CKD powder loaded with dyes (either MB or CR) can be utilized as a filler in the unsaturated polyester, to reduce its production cost and maintain its properties, as will be implemented and discussed later.

**Table 4:** Linear and non-linear parameters for adsorption isotherms models of MB and CR dyes on CKD surface.

Dye	Langmuir model						Freundlich model						
	b (Lmg^-1^)	*Q*_*e*_ (mg/g)	$Q_{max}$(mg/g)	$Q_{max}$(mg/g) (nonlinear fit)	R^2^	R^2^ (nonlinear fit)	R_L_	$K_{f}$(mg/g)	$K_{f}$ (mg/g) (nonlinear fit)	*n*	*n* (nonlinear fit)	R^2^	R^2^ (nonlinear fit)
MB	0.01	38.44	58.43	59.144	0.9962	0.9983	0.305	3.681	2.011	1.701	1.79	0.9939	0.9910
CR	0.01	84.98	123.42	127.28	0.9905	0.9984	0.239	3.327	4.059	1.533	1.66	0.9936	0.9918

**Table 5:** The adsorption capacities for some adsorbents compared to the studied CKD adsorbent.

Adsorbent	Absorbate	Adsorption capacity (mg/g)	References
Walnut shells powder	Methylene blue	25–200	^8^
Fly ash	Congo Red dye	22.12	^13^
Gypsum	Chlorazole yellow and methylene blue	12.85	^21^
Vaterite calcium carbonate	Congo red dye	12.4–16.5	^30^
Orange peel	Congo red dye	18.94	^39^
Hydroxyapatite- (HAP-) Clay Composites	Methylene blue	17.20–20.7	^42^
Crystalline hydroxyapatite (HAP) nanomaterial	Methylene blue	14.27	^43^

## Conclusion

The CKD powder ‘the adsorbent in this study’ was characterized by using the XRF, N_2_ adsorption–desorption BET, FTIR, and SEM tests. The adsorption of both MB and CR dyes on CKD particles` surfaces from the simulated wastewater solution was studied by applying varying experimental conditions such as various mixing contact times, initial concentrations of the dye, temperatures, pH as well various initial doses of the adsorbent. The uptake of such dyes increased linearly with rising temperature, the adsorption contact time (till 120 min), the mass of CKD as well as the pH of the cationic MB dye. On the other hand, the adsorption of dyes decreased with increasing the initial concentration of dye besides the pH of the anionic CR dye. The results revealed that the pseudo-second-order model was well fitted to the kinetic data rather than other applied models, such as the pseudo-first-order and intraparticle diffusion kinetic models. Studying the thermodynamics parameters revealed that the adsorption process was endothermic and spontaneous. Langmuir and Freundlich's adsorption isotherm models were studied linearly and nonlinearly, and the findings showed that the uptake process of MB and CR dyes was followed by both the processes of homogeneous monolayer and heterogeneous multilayer coverage on the outer surface of the CKD powder. Finally, this article pointed out the utilization of one of the industrial hazardous pollutants, i.e., CKD, in the treatment of simulated polluted wastewater, realizing that CKD powder can be a practical adsorbent with little cost for the effective removal of dyes from industrial wastewater achieving an environmental and economical solution for the addressed issues simultaneously at the same time.

## Data Availability

All data generated or analyzed during this study are included in this article.
